# The Demand Supply Steady-State Process-Based Multi-Level Spare Parts Optimization

**DOI:** 10.3390/s21248324

**Published:** 2021-12-13

**Authors:** Jiaju Wu, Huijun Liu, Hongfu Zuo, Zheng Cheng, Yonghui Yang, Yongqi Ma, Linggang Kong

**Affiliations:** 1 College of Civil Aviation, Nanjing University of Aeronautics and Astronautics, Nanjing 210016, China; wujj@caep.cn (J.W.); rms@nuaa.edu.cn (H.Z.); 2 Institute of Computer Application, China Academy of Engineering Physics, Mianyang 621900, China; lhj12uestc@163.com (H.L.); chengz@caep.cn (Z.C.); Yangyh@caep.cn (Y.Y.); myq123456a@caep.cn (Y.M.)

**Keywords:** spare parts, optimization process, multi-level, spare parts support process, demand rate, supply rate, system availability

## Abstract

Spare parts are one of the important components of the equipment comprehensive support system. Spare parts management plays a decisive role in achieving the desired availability with the minimum cost. With the equipment complexity increasing, the price of spare parts has risen sharply. The traditional spare parts management makes the contradiction between fund shortage and spare parts shortage increasingly prominent. Based on the analysis of the multi-echelon and multi-indenture spare parts support model VARI-METRIC (vary multi-echelon technology for recoverable item control, VARI-METRIC), which is widely used by troops and enterprises in various countries, the model is mainly used in high system availability scenarios. However, in the case of low equipment system availability, the accuracy and cost of model inventory prediction are not ideal. This paper proposed the multi-level spare parts optimization model, which is based on the demand-supply steady-state process. It is an analytical model, which is used to solve the low accuracy problem of the VARI-METRIC model in the low equipment system availability. The analytical model is based on the multi-level spare parts support process. The article deduces methods for solving demand rate, demand–supply rate, equipment system availability, and support system availability. The marginal analysis method is used in the model to analyze the spare parts inventory allocation strategy’s current based cost and availability optimal value. Finally, a simulation model is established to evaluate and verify the model. Then, the simulation results show that, when the low availability of equipment systems are 0.4, 0.6, the relative errors of the analytical model are 3.54%, 3.86%, and its costs are 0.52, 1.795 million ¥ RMB. The experiment proves that the inventory prediction accuracy of the analytical model is significantly higher than that of the VARI-METRIC model in low equipment system availability. Finally, the conclusion and future research directions are discussed.

## 1. Introduction

Comprehensive equipment support provides all kinds of necessary supply, repair and maintenance for the normal use of equipment by adopting comprehensive and perfect methods [1]. The equipment integrated support system is mainly composed of information, support equipment, training, on-site technical support, on-site work and spare parts. Spare parts are one of the important aspects of an equipment integrated support system [2]. As the most commonly used inventory, spare parts are necessary for equipment maintenance. The cost of spare parts accounts for a large part of the equipment life cycle cost. Spare parts cost is the main part of equipment maintenance cost, accounting for more than half of the total cost [3]. When the equipment fails to maintain, for no spare parts in stock, the equipment downtime caused by insufficient spare parts will cause huge economic losses. Spare parts management plays a decisive role in achieving the desired availability with minimum cost. The characteristics of spare parts management are as follows:

- First, the demand for spare parts is difficult to predict. There is an intermittent demand pattern among spare parts. They are characterized by some zero demand observation sequences interspersed with occasional non-zero demand [4];
- Second, the quantity and variety of spare parts are usually very large. Even for medium-sized enterprises, there are thousands of different spare parts in the warehouse [5]. It is difficult to formulate a very suitable inventory storage strategy for each spare part;
- Third, to reduce the spare parts scraping risk, we need to minimize inventory. If the inventory strategy is improper, it may lead to serious inventory cost backlog or equipment downtime cost;
- Fourth, the loss of spare parts is closely related to spare parts maintenance. When the corresponding parts of the equipment are broken down, damaged or worn, they need to be replaced by spare parts. Because the quantity and importance of various spare parts in the equipment are different, the importance and assembly quantity of certain spare parts for the equipment and the equipment system are different [6]. Therefore, the equipment use mode and maintenance strategy is an important base for designing the spare parts inventory support system.

A large number of works have studied the general product manufacturing, sales and after-sales supply chain and inventory management, such as the quality and cost optimization control method based on a constant and fuzzy demand strategy [7], the supplier evaluation and recommendation system method based on an analytic hierarchy process and fuzzy reasoning system [8], the supply chain inventory management strategy using intelligent technologies such as machine learning and radiofrequency identification (RFID) [9], and so forth. The supply chain management of general products generally takes the production, sales, use, operation and maintenance of products as a single link. Inventory considers products as a basic unit. Usually, producers or manufacturers carry out inventory management and maintenance guarantees, but not from the perspective of the system. The supply chain management of general products does not consider the impact of multi-level maintenance and supply support systems, the multi-level failure transmission mechanism of the complex equipment structure and different system reliability caused by the particularity of the equipment system operation environment inventory strategy. Therefore, it has limited application in the field of the maintenance and support of complex equipment, especially in the military field.

From the perspective of the life cycle of equipment, spare parts generally do not have substantial consumption during the supply process, as the top-level station has strong maintenance ability and can completely repair all the failure parts. It can be supplemented with the purchasing method even if it is scrapped due to the failure of repair. In this case, the quantity and inventory probability of supply channels will tend to be stable in the long-term maintenance and supply process of spare parts without material consumption, which belongs to the steady process of demand–supply [10]. Demand–supply steady-state process-based multi-level maintenance and supply is a more scientific support mode, which is widely used by various troops or enterprises. In the case of no real consumption, in the spare parts long-term maintenance and supply process, the number of supply channels and inventory probability will tend to be stable [11,12]. Thus, it can be seen that the support station’s inventory allocation problem at all levels under the demand–supply steady-state process condition is in the overall planning of the equipment and its spare parts’ whole life cycle, which is of great significance for the military in controlling and grasping the supporting spare parts needed for new equipment [13].

The research object of this paper is complex equipment with a multi-level structure with a multi-level maintenance and supply support system, such as aircraft, missiles, submarines, aircraft carriers and so on. The equipment may operate under extremely complex working conditions such as high temperature, high voltage, lightning, storm, high salt, low temperature and low pressure, and the availability of the equipment system is very likely to be low. Therefore, it is necessary to predict the multi-level maintenance and supply inventory strategy and inventory cost of spare parts for different availabilities of equipment systems, to ensure low-cost and efficient maintenance in the case of equipment failure. At the same time, the inventory strategy needs to consider the structural level of equipment, maintenance and supply level, transportation time of spare parts, installation quantity of equipment systems to components, influence rate of equipment system failure caused by component failure, line replacement unit (LRU), shop replacement unit (SRU), series-parallel repair, maintenance capacity, maintenance channel, and so forth. For complex equipment, especially weapons, the equipment development party and the equipment use support party jointly realize the maintenance support of equipment through the military–civilian integration mode and pay more attention to the multi-level supply and maintenance support capability under certain equipment system availability and support system availability, to achieve the optimal inventory strategy and cost.

For research on the multi-level spare parts inventory strategy under a demand–supply steady-state process, the current mainstream methods are a metric series model theory, including METRIC, Mod-METRIC, Dyna-METRIC, VARI-METRIC, and so forth [13]. The METRIC [14] model is the basis of this series of models, while the VARI-METRIC model is the final form of the multi-echelon support structure and a multi-indenture spare parts allocation optimization model [15]. The VARI-METRIC model is widely used and accepted. However, many assumptions are made in the process of establishing a model, which leads to the low accuracy of the equipment system at low availability [13]. When the equipment system is under complex and extreme conditions, the availability of the equipment system may be reduced to a certain extent. A high availability of the equipment system cannot be guaranteed at any time. Therefore, the model needs to be improved to ensure the accuracy of an inventory optimization strategy in a high availability of equipment system and to improve the accuracy of the spare parts inventory at low availability. This research aims to improve the VARI-METRIC model’s accuracy at low system availability, which is based on the demand–supply steady-state process. The rest of this paper is organized as follows.

- Section 2 describes the research literature review;
- Section 3 provides a multi-level spare part optimization model based on the demand–supply steady-state process, according to the VARI-METRIC theory, which is called the analytical model. The analytical model includes five parts: multi-echelon and multi-indenture spare parts support, spare parts demand rate, spare parts demand–supply and supply rate, equipment system demand–supply and spare parts optimization solution. Section 3.2 analyses the multi-level spare parts support process. Section 3.3 defines the model’s variables, restrictions and parameters. Section 3.4 deduces the spare parts’ demand rate calculation method. The first level and second level LRU and SRU [16] in the rear warehouse and base I demand rate calculation method are detailed. Section 3.5 describes the multi-level spare parts’ demand–supply models. Section 3.6 deduces a demand–supply solution. LRU and SRU’s spare parts availability and support delay time in the base or rear warehouse are detailed. The whole equipment system availability and the support system availability calculation method are deduced in the paper. Section 3.7 describes the solution process of the model;
- Section 4 describes the model simulation and verification. Section 4.1 establishes the simulation model to evaluate and adjust the multi-level spare parts optimization model based on the demand–supply steady-state process. Section 4.2 describes comparative experiments between the simulation model and the analytical model, and the analytical model and the VARI-METRIC model. The results are analyzed in Section 4.3;
- Section 5 describes the conclusion, some limitations and future expansion of the article;
- Finally, some patents are declared and relevant references are provided.

## 2. Related Research

Silver [17], Kennedy et al. [18], Boylan et al. [4], Syntetos et al. [19,20], Paterson et al. [21], Bacchetti et al. [22], Bakker et al. [23] and Houtum [24] summarized the literature review related to spare parts inventory management, including a spare parts inventory management system, the replacement of spare parts, multi-level problems, the scrap of spare parts, forecasting research and extensions, inventory models considering horizontal transshipment, inventory system deterioration, the system-oriented spare parts inventory model, supply chain forecasting, and so forth [25].

Sherbrooke [14] put forward the METRIC model based on the multi-echelon inventory system spare parts demand law and objective function [25]. Simon [26] simplified the multi-echelon inventory system to obtain the exact solution. Muckstadt [27,28] proposed the MOD-METRIC model, which extended the METRIC model [26]. Hillestad et al. [29,30] further extended the MOD-METRIC model and proposed the Dyna-METRIC model [25]. Graves [15] proposed the VARI-METRIC model, which improved the METRIC model’s expected value of order quantity [25]. Later, Sherbrooke [31,32] improved the VARI-METRIC model by using Graves’ two-parameter approximation method and by considering the lateral supply factor. Angel Diaz et al. [33] and A. Sleptchenko et al. [34] improved the VARI-METRIC model from limited repair capacity. F Costantino et al. [35] extended the VARI-METRIC model from system availability and budget constraints [25]. GJ Van Houtum et al. [36] improved the VARI-METRIC model under system availability constraints. Based on the VARI-METRIC model and the combined actual support requirements, many scholars have improved the model, including considering limited maintenance channels [37,38], series maintenance [39,40,41], horizontal replenishment [42], spare parts weight [43], spare parts sharing [44], imperfect maintenance [45], and part universality [46], and so forth. Spare parts optimization software tools VMETRIC and OPUS [47,48] take it as the core model and algorithm, which are used widely now. The advantages and disadvantages of the multi-level spare parts optimization model are shown in Table 1.

## 3. Demand Supply Steady-State Process-Based Multi-Level Spare Parts Optimization Model

### 3.1. Multi-Level Spare Parts Optimization Model

**Figure 1:**
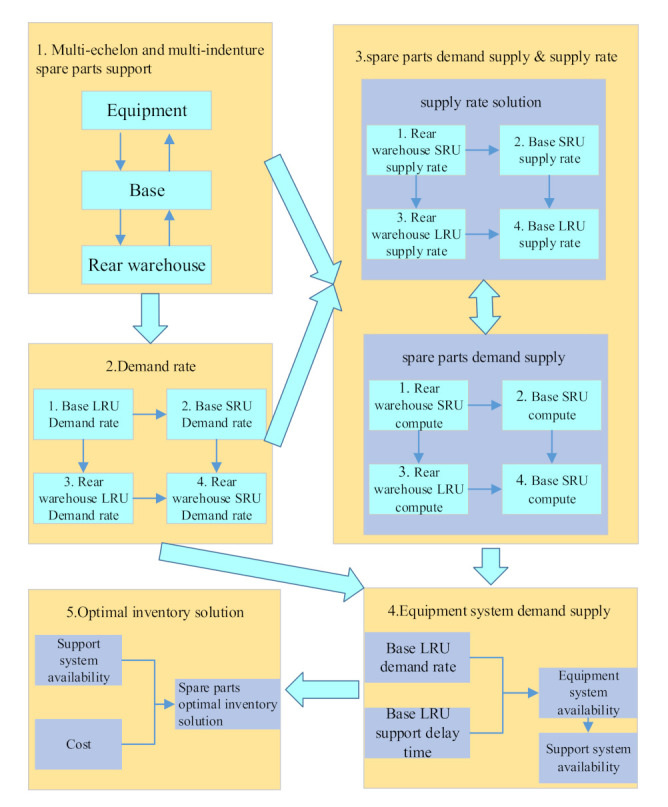
The multi-level spare parts optimization model.

The demand–supply steady-state process-based spare parts optimization model is shown in Figure 1. It includes five modules, which are a multi-echelon and multi-indenture spare parts support process, spare parts demand rate solution, spare parts demand supply and supply rate, an equipment system demand supply and a spare parts optimization solution.

The equipment’s multi-level spare parts support includes multi-echelon and multi-indenture structure support. The demand rate and supply rate of spare parts are the important factors that affect the smooth progress of the whole support process. The demand for spare parts at all levels promotes the whole support process, which makes the number of different spare parts in the whole system gradually reduce. For the supply of spare parts at all levels, the spare parts demand promotes the whole support process. They let the number of different spare parts in the whole system gradually increase. The demand rate and supply rate put the number of spare parts in the whole system into a steady equilibrium state. Once we set the initial number of spare parts at all levels, we can keep the system probability in a stable state, so that the system can operate stably within the controllable range.

**Figure 2:**
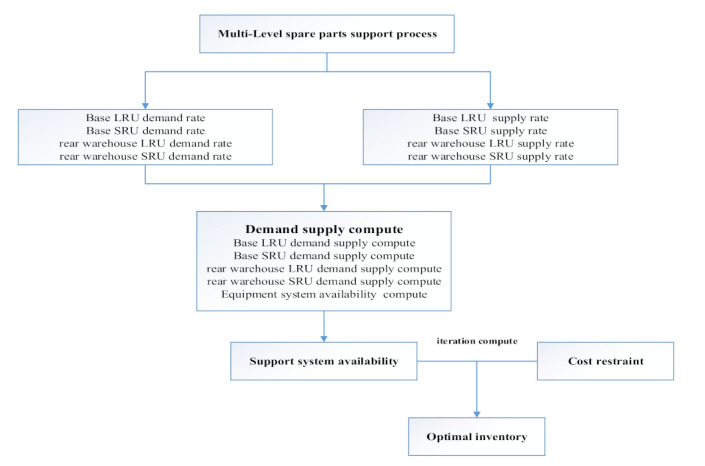
Multi-echelon and multi-indenture spare parts optimization process.

The demand–supply steady-state process-based multi-level spare parts optimization process is an analytical calculation process, which is shown in Figure 2. Firstly, according to the multi-level spare parts support process, each level spare parts demand rate of each support echelon is derived. The order of derivation is the first level spare parts demand rate of the base, the second level spare parts demand rate of the base, the first level spare parts demand rate of the rear warehouse and the second level spare parts demand rate of a rear warehouse. Then, according to the support process and the demand rate of specific support echelon spare parts, the spare parts’ supply rate of each support spare part is derived. The order of derivation is the second level spare parts’ supply rate of the rear warehouse, the first level spare parts supply rate of the rear warehouse, the second level spare parts supply rate of the base and the first level spare parts supply rate of the base. Next, five sub calculation processes of demand–supply are constructed, which are respectively the calculation of spare parts availability and support delay time at the second level of the rear warehouse, the calculation of spare parts availability and support delay time at the first level of the rear warehouse, the calculation of spare parts availability and support delay time at the second level of the base, the calculation of spare parts availability and support delay time at the second level of the base, the first level of base spare parts availability and support delay time calculation and base equipment availability calculation. Then, the availability of the whole equipment support system is calculated according to the five sub calculation processes. Finally, according to the objective function, the maximum availability and the cost limit constraint condition, combined with the marginal analysis method, the spare parts’ optimal inventory at all levels is derived.

### 3.2. Multi-Level Spare Parts Support Process

**Figure 3:**
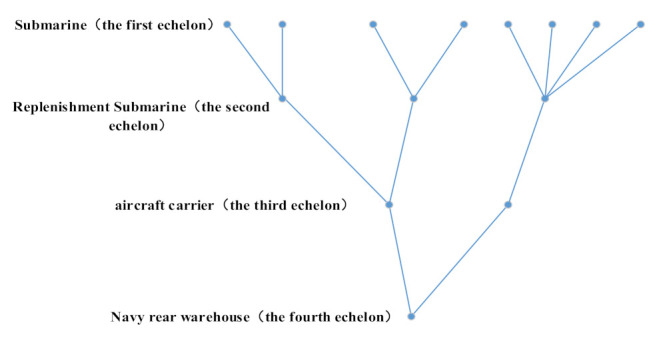
Multi-echelon support structure.

The equipment’s multi-level spare parts support includes multi-echelon maintenance supply level and multi-indenture equipment hierarchy structure support. Multi-echelon spare parts support refers to the support level corresponding to the equipment maintenance level. For the number of spare parts, we are usually interested not only in the required number of spare parts for each base but also in the spare parts storage required for the rear warehouse of each support base. Here, the base is called the first echelon and the rear warehouse is called the second echelon. For example, in most cases, the navy is considered a two-stage supply system. Sometimes, there are more echelons. For example, to support the deployed submarines, each submarine (first echelon) has some spare parts. At the same time, some spare parts are stored in the second-class supply ship, which can enter the submarine regularly. These facilities are supported by the third-echelon home port. Finally, there is the fourth echelon of the naval rear warehouse. Figure 3 shows a typical submarine multi-echelon support structure, which is composed of eight first-echelon stations, three second-echelon stations, two third-echelon stations and one fourth-echelon station.

**Figure 4:**
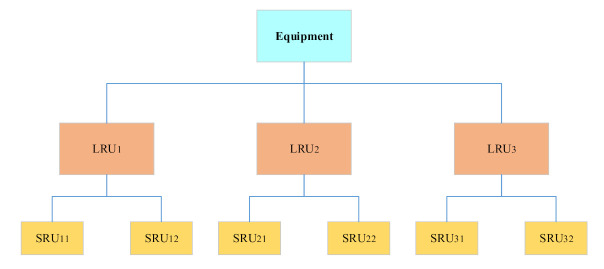
Multi-indenture structure.

The multi-indenture structure describes how the whole equipment system is composed and how the spare parts are assembled, as shown in Figure 4. LRU is the first level of spare parts. When the first level spare parts are disassembled at the maintenance point, it is found that the fault of the LRU is caused by the sub-component of the LRU, which is replaced. Here, the sub-component is called the shop replacement unit (SRU) [16], also known as the second-level spare parts. All components that can be directly removed from LRU are called SRU. Similarly, when the SRU continues to be repaired in the workshop, the SRU is disassembled. It is found that the failure is caused by the sub-components of the SRU, which can be called sub shop replacement unit (SSRU), also known as the third level spare parts. By analogy, all levels of spare parts have their level of spare parts name.

**Figure 5:**
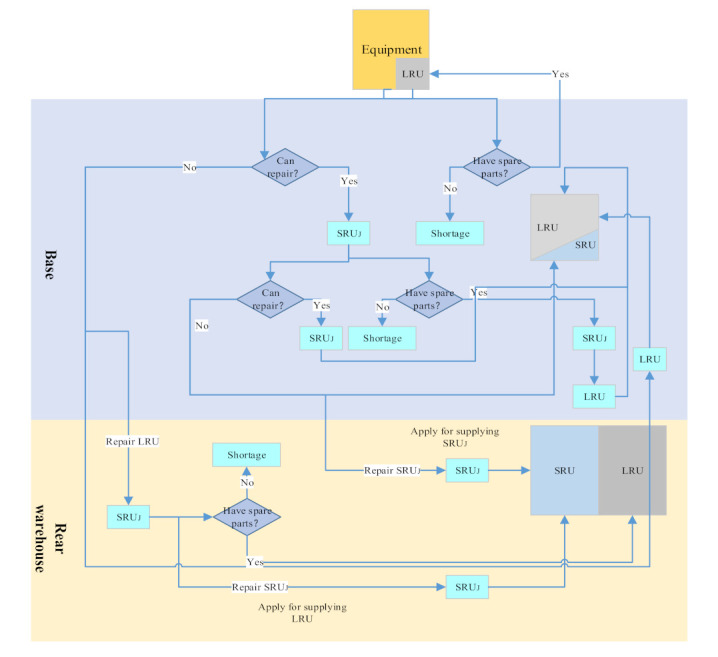
Multi-level spare parts support process.

This paper takes the two-level and two-level equipment systems as an example to describe the multi-level spare parts support process, as shown in Figure 5. For the two-level support system, the base is the first-level support site. The rear supply warehouse is the second-level support site. For the two-level equipment structure, the first level spare parts are named by LRU, and the second level spare parts corresponding to LRU are named by SRU. Each support station has a corresponding spare parts storage space, which stores a certain number of LRU and SRU, respectively.

When the equipment is in normal operation, the whole equipment will stop due to the failure of one LRU. At this time, the base uses the methods of replacing lost efficacy parts and repairing failed parts to deal with the failed LRU. For the failed LRU, it should be transported to the base first. If the LRU storage space is in stock at this time, the LRU should be replaced immediately to complete the equipment repair. If the storage space is not in stock at this time, a spare parts shortage event of the LRU will occur. At the same time, due to the limited maintenance capacity of the base, the failed LRU will be maintained in the base with a certain probability. If it is not maintained in the base, the relevant spare parts manager will send the failed LRU to the rear warehouse for maintenance. He will make a replenishment application for the LRU in the rear warehouse. If the LRU storage space in the rear warehouse is in stock, the relevant spare parts manager will send the failed LRU to the rear warehouse for maintenance. Then, the spare part is added to the base LRU storage space. For the LRU maintained in the base, if it is found that the LRU failure is caused by one of its SRU failures, the failure SRU is also treated in a way similar to LRU. The failure SRU spare parts are replaced and repaired. For the failed SRU, if the SRU spare parts storage space is in stock at this time, the SRU will be replaced immediately to complete the failed LRU repair. Meanwhile, the relevant spare parts staff will send the successfully repaired LRU to the corresponding SRU spare parts storage space in the base. However, if there is no inventory in the corresponding SRU spare parts storage space, a spare parts shortage event will occur. At the same time, due to the limited maintenance capacity of the base, the SRU has a certain probability of repair in the base. After a certain maintenance time, the failed SRU is successfully repaired. The relevant spare parts staff will send the repaired SRU to the SRU spare parts storage space in the base. If the failed SRU cannot be repaired in the base, the relevant spare parts manager will send it to the directly corresponding upper-level site. Failed SRU will be repaired in the rear warehouse. He will make a replenishment application for the SRU in the rear warehouse. If the SRU spare parts storage space in the rear warehouse has inventory, the spare parts will be added to the SRU storage space of the base.

For the rear warehouse, we assume that it has strong reparability and the repair probability for all spare parts is 1. For the failed LRU transported from the base, the rear warehouse will check the failed LRU and find out the SRU, which caused the LRU failure. For the failed SRU, it will be repaired in the rear warehouse for a while. After the repair is successful, a relevant person will assemble the repaired SRU to its LRU. At this time, the whole LRU is repaired successfully. Then, the relevant spare parts staff will send the repaired LRU to the corresponding LRU spare parts storage space of the rear warehouse. For the failed SRU transported from the base, the rear warehouse will directly repair the failed SRU. After a period of maintenance, the repair is successful. Then, the repaired SRU will be directly sent to the corresponding SRU spare parts storage space of the rear warehouse. Due to the high priority of SRU spare parts and LRU spare parts shortage events in the base, once the repaired spare parts or the spare parts in the rear warehouse are generated, the two spare parts shortage events will be met the first time.

### 3.3. Model’s Variables, Restrictions & Parameters

The variables of the optimization model are the maintenance support echelons and the equipment’s structural indentures. To simplify the model calculation, this paper adopts two-level maintenance support to meet most maintenance situations, including base-level maintenance and rear warehouse level maintenance (0 = rear warehouse). The hierarchical structure of equipment adopts the typical three-level maintenance support mode which are equipment system, LRU, SRU (0 = equipment system).

To simplify the modeling process and consider the actual situation, this paper formulates some restrictions on the support process.

Condition 1: All LRUs are series in the equipment, and all LRU’s SRUs are in series. In this case, once one LRU fails, the whole equipment system will stop running.

Condition 2: The failure of LRU is only caused by an SRU. The failure probability is q_IJ_. Here, the multiple second-tier spare parts failure which will cause the failure of corresponding first-level spare parts has not been considered.

Condition 3: The maintenance probability of the rear warehouse is 1.

Condition 4: The equipment system maintenance channel is unlimited [30]. There is no need to queue maintenance spare parts.

Condition 5: All kinds of spare parts’ failure-free operation time are subject to the exponential distribution [13]. This assumes is used to calculate the failure-free working time of spare parts at each level.

Condition 6: All kinds of spare parts’ demand rates are subject to Poisson distribution [13,33].

Condition 7: The spare parts demand–supply strategy of each grade site is (S-1, S) inventory strategy in (s, S) inventory strategy [25]. (s, S-1) inventory strategy is continuous inventory [50,51]. Once the inventory level is less than s, the maintenance station will order immediately. The order quantity makes the inventory level at the order time reach S. Otherwise, no order is made. Therefore, the inventory strategy of (s, S-1) in this paper is that, once there is a good demand, we order the goods to the manufacturer once regardless of whether there are any goods in the inventory. If there are any goods, we submit one delivery. Otherwise, there is a shortage of goods.

The following parameters are required for the spare parts optimization process, which is shown in Table 2. I is used as base number 1, 2, 3, …, b (0 = rear warehouse) according to the convention. J is used to represent item No. 1, 2, 3, …, n (0 = LRU of the first level spare parts).

### 3.4. Multi-Level Spare Parts Demand Rate

(1) The base I’s first level spare parts of LRU demand rate.

By analyzing the whole multi-echelon and multi-indenture spare parts support process, it can be seen that the equipment shutdown is caused by one of the first level spare parts LRU failures. For example, once the engine of the aircraft stops running, the whole aircraft will stop running. To reduce the equipment downtime as much as possible, to reduce the unpredictable loss caused by equipment downtime, the whole support system will immediately generate a demand for the first level spare LRU in the base. So, the failure rate of the first level spare LRU is equivalent to the demand rate of the first level spare LRU in base I.

(1) $$\lambda_{I0}=\frac{W_{T1}\cdot Z_{0}\cdot N_{I}}{{\mathrm{MTBF}}_{0}}\;.$$

(2) The base I second level spare parts SRU demand rate.

The second level spare parts SRU demand rate of base I is for the failure LRU with a certain probability r_I0_, which can be maintained in the base. The probability q_IJ_ is caused by the failure of SRU_J_. For the failed SRU_J_, to repair the LRU as soon as possible and reduce the downtime of the equipment, there is an immediate demand for base I to change the SRU_J_. Therefore, the failure rate of the SRU_J_ found by the LRU failure in base I is equivalent to the demand rate of base I for SRU_J_.

(2) $$\lambda_{\mathrm{IJ}}=\lambda_{I0}\cdot R_{I0}\cdot q_{\mathrm{IJ}}.$$

For q_IJ_, LRU is the probability of failure due to SRU_J_ failure. MTBF_0_ is the average time between LRU failures. MTBF_J_ is the average failure interval of SRU_J,_ which belongs to LRU. Since the LRU failure must be caused by one SRU_J_ failure,

(3) $$q_{\mathrm{IJ}}=\frac{{\mathrm{MTBF}}_{0}}{{\mathrm{MTBF}}_{J}}$$

(3) The first level spare parts LRU demand rate in the rear warehouse.

The LRU demand in the rear warehouse is caused by the LRU failure, which cannot be maintained in the base and be sent back to the warehouse. Meanwhile, the LRU requirement modification is proposed to the warehouse. For the rear warehouse, it should guarantee b bases at the same time. So, it should sum up the demand for all b bases.

(4) $$\lambda_{00}=\sum_{I=1}^{b}\lambda_{I0}\left(1-r_{I0}\right).$$

(4) The second level spare parts SRU demand rate in the rear warehouse.

The SRU_J_ requirement for the rear warehouse is generated by two parts. The first part is generated by the SRU_J_ supplementary application, which is sent to the rear warehouse by the SRU_J_ and it cannot be repaired on all bases. The second part is caused by the LRU being sent to the rear warehouse for repair, which cannot be repaired on all b bases. The SRU_J_ requirement is the SRU_J_ application for the rear warehouse, in which the failed LRU detects the corresponding failed SRU_J_ during the maintenance of the rear warehouse.

(5) $$\lambda_{0I}=\sum_{I=1}^{b}\left[\lambda_{\mathrm{IJ}}\left(1-r_{\mathrm{IJ}}\right)\right]+\lambda_{00}\cdot q_{\mathrm{IJ}}.$$

(5) Demand rate solution

**Figure 6:**
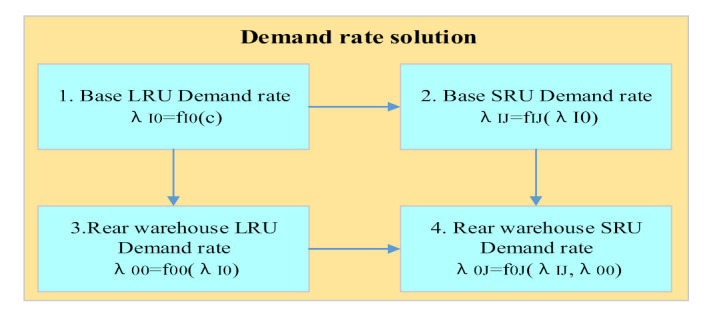
Demand rate correlation and solution sequence diagram.

The specific solving order of each demand rate and the relationship between them are shown in Figure 6. We take the data given by the equipment system in advance as constant c. First, we calculate the LRU demand rate of the base according to the known information, which is not associated with other demand rates. Second, according to Formulas (2) and (4), the SRU demand rate in the base and LRU in the rear warehouse are functions of the spare parts demand rate. So, the SRU in the base and LRU in the rear warehouse demand rate can be calculated at the same time. In the third step, according to Formula (5), the SRU demand rate of the rear warehouse is a common function of the SRU demand rate in the base and the LRU in the rear warehouse. So, the SRU demand rate of the rear warehouse can be solved by combining the two demand rates.

### 3.5. Multi-Level Spare Parts Demand-Supply Model

**Figure 7:**
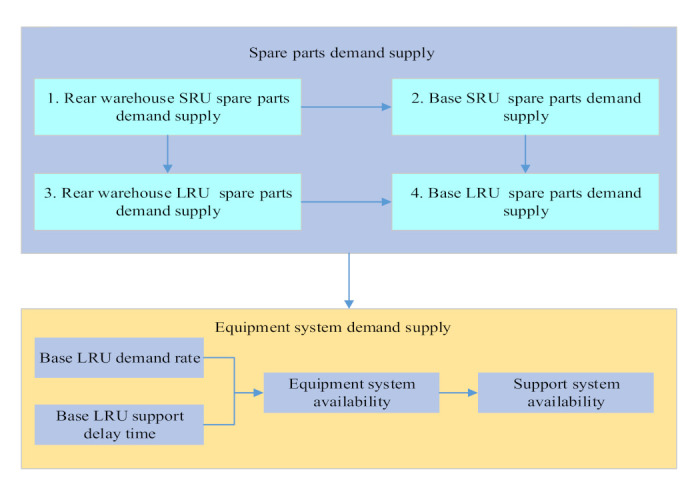
Multi-level spare parts demand-supply model.

According to the multi-echelon and multi-indenture spare parts support process based on the demand–supply steady-state process, we can divide the whole multi-level spare parts demand–supply into two parts, which are spare parts demand–supply and equipment system demand–supply. The multi-level spare parts demand–supply model is shown in Figure 7. Other multi-level spare parts’ demand–supply can be expanded in the same way.

The three decisive variables of the multi-level spare parts demand–supply model are system state variables, demand quantity and supply rate. The system status variable represents the current inventory of the spare parts storage point at the current level. Its status value can be reduced due to demand or increased due to supply. Demand rate is the velocity with which the system generates a spare parts demand when it is in a special state. Due to the system demand rate, it transfers the state from 1 to 2. The inventory of state 2 is reduced by 1 compared with state 1. Supply rate is the velocity at which the system receives a spare parts supply when it is in a state. Due to the system supply rate, the system changes the state from 3 to 4. The inventory of state 4 increases by 1 relative to state 3.

**Figure 8:**
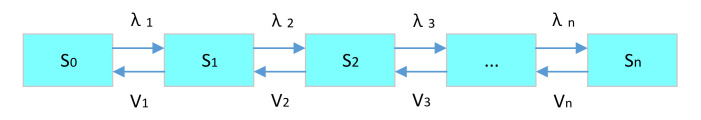
Multi-level demand-supply inventory status transition diagram.

For the state variable, the maximum value is the initial inventory quantity. The minimum value is the negative value of the system’s maximum shortage. The specific demand–supply inventory status transition is shown in Figure 8.

We define the initial state as S_0_, the next state as S_1_ … and so on, the last state as S_n_, where the inventory of the latter state is 1 larger than that of the previous state.

For the maximum number of spare parts shortage S_n_, if it is the second level SRU spare parts, the maximum number of spare parts shortage is 1. The spare parts initial number in the first level LRU spare parts storage point is the assembling number. Here in one LRU, only one SRU is assembled. If n is assembled, then the initial number of spare parts multiplies n. If it is the first level LRU spare parts, the maximum shortage is the LRU spare parts assembly quantity in a piece of equipment add the LRU spare parts storage point to the initial quantity of the next support level.

When the system status is in spare parts shortage, the system stops running. So, the maximum shortage is the consumption of all the LRUs in the system.

The model assumes that the time tends to infinity and the system is in the limit stationary state. At this time, although the system state changes randomly, their state probability no longer changes with time. The state probability P_i_ of S_i_ state means the average time that the system is in S_i_ state relative to all other states. So, the state probability P_i_ is constant.

For the state S_0_ and the Gormorkov equation in the above demand–supply model, the following equation can be obtained:

(6) $$\frac{{\mathrm{dP}}_{0}}{\mathrm{dt}}=V_{1}P_{1}-\lambda_{1}P_{0}.$$

Because $P_{0}$ is a constant, the above formula is 0, so $V_{1}P_{1}=\lambda_{1}P_{0}.$

For the state S1 and Gormorkov equation in the above demand–supply model, the following equation can be obtained:

(7) $$\frac{{\mathrm{dP}}_{0}}{\mathrm{dt}}=\lambda_{1}P_{0}+V_{2}P_{2}-\lambda_{2}P_{1}-V_{1}P_{1}.$$

Because P_1_ is a constant, the above formula is 0, so $V_{2}P_{2}=\lambda_{2}P_{1}$. By analogy, the following equations can be obtained:

(8) $$\left\{\begin{array}{c} \lambda_{1}P_{0}=V_{1}P_{1}\\ \lambda_{2}P_{1}=V_{2}P_{2}\\ \begin{array}{c} \lambda_{3}P_{2}=V_{3}P_{3}\\ \ldots \\ \lambda_{n}P_{\left(n-1\right)}=V_{n}P_{n} \end{array}\; \end{array}\right..$$

Since P_i_ is the state probability of each state, the sum of the probabilities of all states is 1.

(9) $$\sum_{i=1}^{n}P_{i}=1\;.$$

By solving Equations (8) and (9), the state probability of each state system state can be obtained.

(10) $$\left\{\begin{array}{c} P_{0}=\frac{1}{1+\frac{\lambda_{1}}{V_{1}}+\frac{\lambda_{1}\lambda_{2}}{V_{1}V_{2}}+\ldots +\frac{\lambda_{1}\lambda_{2}\ldots \lambda_{n}}{V_{1}V_{2}\ldots V_{n}}}\\ P_{1}=\frac{\lambda_{1}}{V_{1}}P_{0}\\ \begin{array}{c} P_{2}=\frac{\lambda_{1}\lambda_{2}}{V_{1}V_{2}}P_{0}\\ \ldots \\ P_{n}=\frac{\lambda_{1}\lambda_{2\ldots }\lambda_{n}}{V_{1}V_{2}\ldots V_{n}}P_{0} \end{array} \end{array}\;\right..$$

According to the state probability of each state, the expected shortage number can be easily obtained:

(11) $$\mathrm{EBO}=\sum_{s_{k}<0}s_{k}P_{k},$$

and the availability is:

(12) $$A=\sum_{s_{k}\geq 0}P_{k},$$

If the spare parts’ failure-free working time obeys exponential distribution. The probability of failure has nothing to do with the starting point of time, but only with the length of time. Therefore, if λ_1_ = λ_2_ = … = λ_n_ = λ and the spare parts’ failure-free working time obeys exponential distribution, the expected spare parts support delay time can be derived as follows:

(13) $$\mathrm{dT}=\frac{\mathrm{EBO}}{\lambda }\;.$$

**Figure 9:**
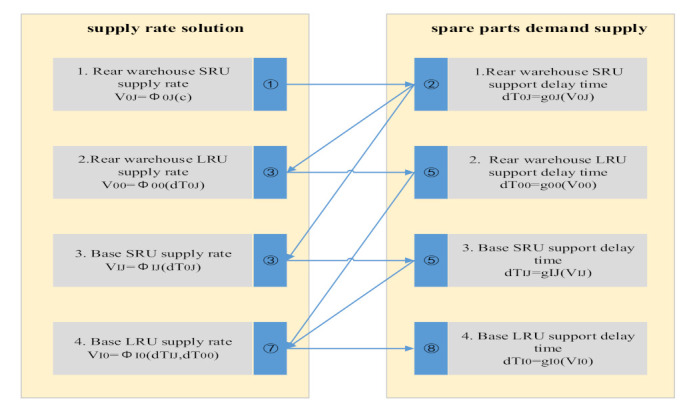
Correlation and solution sequence of supply rate and support delay time.

According to the demand–supply model, we only need to know the spare parts’ initial number and the maximum shortage number in the system state, the demand rate and supply rate between each state transition. We can get the expected shortage number and the availability of spare parts. If the research spare parts’ failure-free working time obeys the exponential distribution, then we can get the expected spare parts to support the delay time. The failure-free working time of the first level spare parts LRU and the second level spare parts’ SRU obeys exponential distribution, then the above three kinds of data can be obtained by establishing spare parts, demand–supply model. The correlation and solution sequence of supply rate and support delay time are shown in Figure 9.

**Figure 10:**
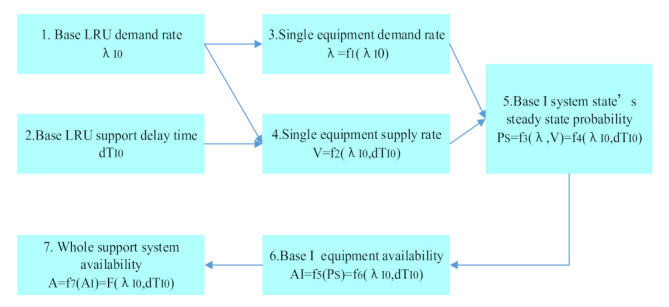
Solution sequence diagram of the whole support system availability.

The multi-level spare parts demand–supply model integrates the multi-echelon and multi-indenture spare parts support process, demand rate solution, spare parts demand supply and supply rate solution content. It solves the steady-state probability of equipment system state and base equipment availability. Finally, we can extend it to all bases of the whole support system, to solve the most important equipment efficiency index in the spare parts system. That is the whole support system’s availability. The specific calculation sequence of support system availability is shown in Figure 10.

### 3.6. Multi-Level Spare Parts Demand-Supply Model Solution

(1) The second level spare parts SRU availability and support delay time in the rear warehouse.

**Figure 11:**
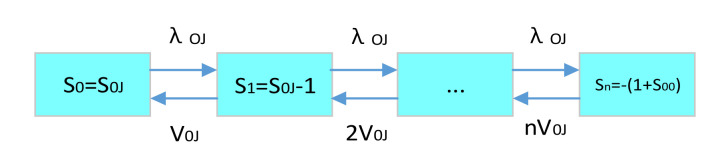
SRU spare parts demand-supply status transition diagram.

According to the multi-level and multi-level spare parts’ supply process, for the spare parts storage point of the second level spare parts SRU in the rear warehouse, the base generates a supply application for the SRU in the rear warehouse due to the local non-repairable SRU. The application rate is the demand rate of the SRU in the rear warehouse. As long as an SRU demand is generated for the rear warehouse at this rate, the number of spare parts in the SRU spare parts storage point in the rear warehouse will be one less. When the inventory of the SRU spare parts storage point in the rear warehouse is reduced to 0, if there is still a demand application for the SRU although the rear warehouse cannot meet its demand application, a shortage will occur. At this time, once the spare parts storage point obtains the spare parts, it will first meet the application for the shortage of modified spare parts. When the storage quantity shortage of SRU spare parts in the rear warehouse is the initial storage quantity of LRU spare parts in the rear warehouse, all the SRUs in the whole supply system (i.e., base level and rear warehouse level) are consumed, that is, all the SRUs are in failure status. At this time, once another demand for the SRU is generated, the warehouse of the party is no longer able to meet the requirements of such spare parts, and the whole equipment stops running immediately. The maximum shortage of the SRU spare parts storage point in the rear warehouse is—(1 adds the initial number of spare parts in the first level spare parts LRU storage point in the rear warehouse). For the supply of SRU spare parts storage point in the rear warehouse, because we assume that the system is an unlimited maintenance channel if n spare parts in the system are in a failure state, the supply rate of the system is n multiplied by that of a single maintenance channel. The specific SRU spare parts’ demand–supply status transition diagram of the rear warehouse is shown in Figure 11.

According to the SRU spare parts demand and supply module of the rear warehouse, as well as Formulas (8) and (9), the steady-state probability of the existing inventory status of each SRU spare parts storage point of the rear warehouse can be deduced as follows:

(14) $$\left\{\begin{array}{c} P_{S_{0}}^{0J}=\frac{1}{1+\sum_{i=1}^{n}\frac{\lambda_{0J}^{i}}{i!V_{0J}^{i}}}\\ P_{S_{i}}^{0J}=\frac{\lambda_{0J}^{i}}{i!v_{0J}^{i}}P_{S_{0}}^{0J}(i>0) \end{array}\right.\;.$$

Here, $n=S_{0J}+S_{00}+1$. Then, according to Formulas (11)–(13), the expected shortage of SRU spare parts in the rear warehouse can be calculated in sequence as follows:

(15) $${\mathrm{EBO}}_{0J}=\sum_{s_{i}<0}s_{i}P_{S_{i}}^{0J}.$$

The availability of SRU spare parts in the rear warehouse is as follows:

(16) $$A_{0J}=\sum_{s_{i}\geq 0}P_{S_{i}}^{0J}.$$

The delay time of SRU spare parts support in the rear warehouse is as follows:

(17) $${\mathrm{dT}}_{0J}=\frac{\mathrm{EB}0_{0J}}{\lambda_{0J}}.$$

(2) Spare parts availability and support delay time of the first level spare parts LRU in the rear warehouse.

A local irreparable LRU generates a supply LRU application in the rear warehouse. The application rate is the LRU demand rate of the rear warehouse. As long as an LRU demand is generated for the rear warehouse at this rate, the spare parts inventory in the rear warehouse will be reduced to one. When the LRU spare parts inventory in the rear warehouse storage point is reduced to 0, if there is a demand application of the LRU, the rear warehouse cannot meet the demand application. Once the spare parts storage point gets the spare parts, it will first meet the demand application of spare parts shortage. However, once the LRU spare parts shortage quantity in the rear warehouse is the initial LRU spare parts storage quantity, all the SRUs in the whole supply system (i.e., base level and rear warehouse level) are consumed. All the LRUs are in a failure state. At this time, once there is another LRU demand, the rear warehouse is no longer able to meet the spare parts requirements. The whole equipment stops working immediately. The LRU spare parts maximum shortage in the rear warehouse storage points is—(1 adds the initial spare parts quantity of the base first level LRU storage points). For the LRU spare parts supply in the rear warehouse storage point, it is assumed that the system is an infinite maintenance channel. So, if n spare parts are in a failure state, the system supply rate is n times that of the single maintenance channel.

**Figure 12:**
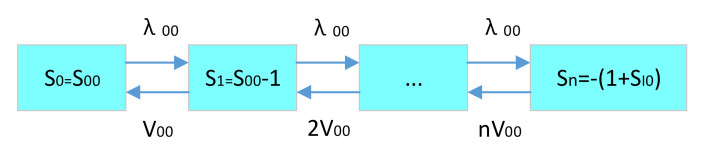
LRU spare parts demand–supply status transition diagram.

The LRU spare parts demand–supply status transition diagram of the rear warehouse is shown in Figure 12.

According to the LRU spare parts demand–supply state transition diagram of the rear warehouse, as well as Formulas (8) and (9), each LRU spare parts steady-state probability in the existing inventory state in the rear warehouse storage point can be deduced as follows:

(18) $$\left\{\begin{array}{c} P_{S_{0}}^{00}=\frac{1}{1+\sum_{i=1}^{n}\frac{\lambda_{00}^{i}}{i!V_{00}^{i}}}\\ P_{S_{i}}^{00}=\frac{\lambda_{00}^{i}}{i!V_{00}^{i}}P_{S_{0}}^{00}\;(i>0) \end{array}\right.\;.$$

Here, $n=S_{00}+S_{I0}+1$. Then, according to the Formulas (11)–(13), the expected shortage of LRU spare parts in the rear warehouse can be calculated as follows:

(19) $$\mathrm{EB}0_{00}=\sum_{s_{i}<0}s_{i}P_{S_{i}}^{00}.$$

The availability of LRU spare parts in the rear warehouse is as follows:

(20) $$A_{00}=\sum_{s_{i}\geq 0}P_{S_{i}}^{00}.$$

The delay time of LRU spare parts support in the rear warehouse is as follows:

(21) $${\mathrm{dT}}_{00}=\frac{\mathrm{EB}0_{00}}{\lambda_{00}}.$$

(3) The second level spare parts SRU availability and support delay time in the base.

According to the multi-level and multi-level spare parts supply process, for the spare parts storage point of the second level spare parts SRU of the base, the spare parts application for the failed SRU caused by repairing the LRU in the base. The application rate is the demand rate of the base SRU. As long as an SRU demand is generated for the base at this rate, the numbers of spare parts in the SRU spare parts storage point of the base will be one less. When the inventory of the SRU spare parts storage point of the base is reduced to 0, if there is still a demand application for the SRU, the base will produce a shortage although it cannot meet its demand application. Once the spare parts storage point obtains the spare parts, it will first meet the application for the shortage of modified spare parts. However, once the shortage of SRU spare parts storage quantity to the base is the initial spare parts storage quantity of base-level LRU, all SRUS at this level, that is, base level, will be consumed. That is, all SRUs are in failure status. At this time, once another demand for the SRU is generated, the whole equipment will stop running immediately because the base is no longer able to meet the requirements of such spare parts. Therefore, the maximum shortage of SRU spare parts storage point of the base is—(1 + the initial number of spare parts of the first level spare parts LRU storage point of the base). For the supply of SRU spare parts storage points in the base, because we assume that the system is an unlimited maintenance channel if n spare parts in the system are in a failure state, the supply rate of the system is n times that of a single maintenance channel.

**Figure 13:**
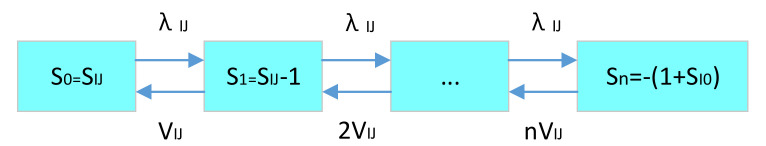
Demand supply status transition diagram of base SRU.

The status transition diagram of SRU spare parts demand and supply in the base is shown in Figure 13.

According to the SRU spare parts’ demand–supply state transition diagram in the base, as well as Formulas (8) and (9), the SRU steady-state probability of the existing inventory state in each base storage point can be deduced as follows:

(22) $$\left\{\begin{array}{c} P_{S_{0}}^{\mathrm{IJ}}=\frac{1}{1+\sum_{i=1}^{n}\frac{\lambda_{\mathrm{IJ}}^{i}}{i!V_{\mathrm{IJ}}^{i}}}\\ P_{S_{i}}^{\mathrm{IJ}}=\frac{\lambda_{\mathrm{IJ}}^{i}}{i!V_{\mathrm{IJ}}^{i}}P_{S_{0}}^{\mathrm{IJ}}\;(i>0) \end{array}\;\right..$$

Here, n = S_IJ_ + S_I0_ + 1. Then, according to Formulas (11)–(13), the expected shortage of SRU in the base can be calculated as follows:

(23) $$\mathrm{EB}0_{\mathrm{IJ}}=\sum_{s_{i}<0}s_{i}P_{S_{i}}^{\mathrm{IJ}}.$$

The availability of SRU in the base is as follows:

(24) $$A_{\mathrm{IJ}}=\sum_{s_{i}\geq 0}P_{S_{i}}^{\mathrm{IJ}}.$$

The delay time of base SRU support is as follows:

(25) $${\mathrm{dT}}_{\mathrm{IJ}}=\frac{\mathrm{EB}0_{\mathrm{IJ}}}{\lambda_{\mathrm{IJ}}}.$$

(4) Spare parts’ availability and support delay time of the base first level spare parts LRU.

For the spare parts’ storage point of the base first level spare parts LRU, the base is the first support level. The equipment generates LRU spare parts application, which is met by the base LRU spare parts storage point. The application rate is the base LRU demand rate. As long as an LRU demand is generated for the base at this rate, the SRU spare parts inventory of the base storage point will be reduced by one. When the inventory of the base LRU spare parts storage point is reduced to 0, if there is a demand application for the LRU, the equipment will stop working immediately due to no LRU supply to the equipment. Therefore, the maximum shortage of base LRU spare parts storage points is 1. For the supply of base LRU spare parts storage points, we assume that the system is an infinite maintenance channel. So, if n spare parts are in a failure state, the system supply rate is n multiples of the single maintenance channel.

**Figure 14:**
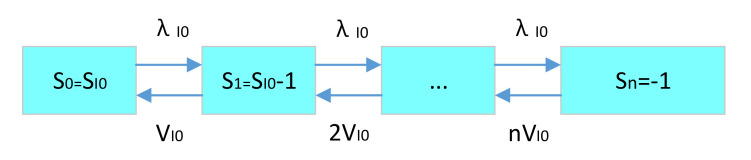
Demand–supply status transition diagram of base LRU.

The specific status transition diagram of LRU spare parts’ demand–supply in the base is shown in Figure 14.

According to the LRU spare parts’ demand–supply status transition diagram of the base, as well as Formulas (8) and (9), the LRU spare parts steady-state probability of the existing inventory status in each base storage point can be deduced as follows:

(26) $$\left\{\begin{array}{c} P_{S_{0}}^{I0}=\frac{1}{1+\sum_{i=1}^{n}\frac{\lambda_{I0}^{i}}{i!V_{I0}^{i}}}\\ P_{S_{i}}^{I0}=\frac{\lambda_{I0}^{i}}{i!V_{I0}^{i}}P_{S_{0}}^{I0}\;(i>0) \end{array}\;\right..$$

Here, $n=S_{I0}+1$. Then, according to Formulas (11)–(13), the expected shortage of LRU spare parts in the base can be calculated as follows:

(27) $$\mathrm{EB}0_{I0}=\sum_{s_{i}<0}s_{i}P_{S_{i}}^{I0}.$$

The availability of LRU in the base is as follows:

(28) $$A_{I0}=\sum_{s_{i}\geq 0}P_{S_{i}}^{I0}.$$

The delay time of base LRU support is as follows:

(29) $${\mathrm{dT}}_{I0}=\frac{\mathrm{EB}0_{I0}}{\lambda_{I0}}\;.$$

(5) Equipment system availability

**Figure 15:**
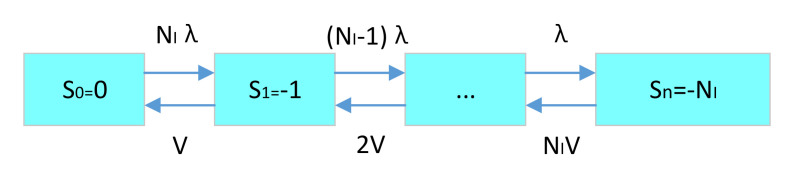
Base I equipment system demand–supply state transition diagram.

Assuming that the system has deployed equipment in the base, the specific demand–supply state transition diagram of the base I equipment system is shown in Figure 15.

The S_0_ = 0 indicates that N_I_ equipment of base I is working normally at this time. S_1_ = −1 means that one piece of equipment of base I stops working and other pieces of equipment are working normally. By analogy, S_n_ = −N_I_ means that all N_I_ pieces of equipment of base I stop working and the whole equipment system is paralyzed. According to the equipment system demand–supply module, we can deduce n = N_I_.

The λ represents the demand rate of a piece of equipment.

(30) $$\lambda =\sum_{m=1}^{z_{0}}\lambda_{I0_{m}\;}.$$

Among them, Z_0_ represents the LRU assembly quantity on one piece of equipment. A single equipment demand rate is the sum of LRU requirements of all bases. Here, m represents the number of LRUs in a device. The demand rate from state S_0_ to state S_1_ is that the demand generated by N_I_ working equipment with rate λ. S_0_, the demand rate at this time is N_I_λ. The demand rate of conversing state S_1_ to state S_2_ is the demand generated by N_I_−1 equipment working normally at the same time. So, the demand rate is (N_I_−1)λ. By analogy, the demand rate from state S_n−1_ to state S_n_ is the demand generated by only one piece of equipment in normal operation, so the demand rate is λ. V represents the supply rate of individual equipment.

(31) $$V=\frac{1}{\mathrm{MT}}\;.$$

Here, MT represents the average maintenance time of a single piece of equipment.

(32) $$\mathrm{MT}=\sum_{m=1}^{z_{0}}\left(q_{I0_{m}}\cdot {\mathrm{dT}}_{I0_{m}}\right).$$

Here, $q_{I0_{m}}$ is the fault location probability. It is the LRU_m_ failure probability causing the equipment to stop working.

(33) $$q_{I0_{m}}=\frac{\lambda_{I0_{m}}}{\sum_{m=1}^{z_{0}}\lambda_{I0_{m}}}.$$

Since the equipment system has been assumed to be an infinite maintenance channel, the supply rate N_I_ is that the equipment stopping operation is supplied at rate V when the state S_n_ changes to the state S_n−1_. So, the supply rate is N_I_V. When the state S_n__−__1_ is changed to the state S_n−2_, the supply rate N_I_−1 is the failing equipment supplied at rate V. So the supply rate at this time is (N_I_−1)V. By analogy, when the supply rate from state S_1_ to state S_0_, there is only one shutdown equipment to supply. So, the supply rate at this time is V.

Therefore, according to the whole equipment system of base I, and Formulas (8) and (9), the steady-state probability of each base I equipment system state can be deduced as follows:

(34) $$\left\{\begin{array}{c} P_{S_{0}}^{\;}=\frac{1}{1+\sum_{i=1}^{N_{I}}\frac{N_{I}!\lambda^{i}}{i!\left(N_{I}-i\right)!V^{i}}}\\ P_{S_{i}}^{\;}=\frac{N_{I}!\lambda^{i}}{i!\left(N_{I}-i\right)!V^{i}}\;(i>0) \end{array}\right..$$

In addition to the status S_0_, the rest of the status have downtime pieces of equipment in the base. So, the availability is calculated from the unavailability. The base I equipment unavailability $\bar{A_{I}}$ is as follows:

(35) $$\bar{A_{I}}=\sum_{i=1}^{N_{I}}\left(P_{S_{i}}^{\;}\cdot \frac{i}{N_{I}}\right).$$

According to the unavailability, the base I equipment availability can be deduced here to calculate:

(36) $$A_{I}=1-\sum_{i=1}^{N_{I}}\left(P_{S_{i}}^{\;}\cdot \frac{i}{N_{I}}\right).$$

The problem is extended to the whole support system. There are b bases, so the availability A is as follows:

(37) $$A=\frac{\sum_{l=1}^{b}\left(A_{\mathrm{Il}}\cdot N_{\mathrm{Il}}^{\;}\right)}{\sum_{l=1}^{b}N_{\mathrm{Il}}^{\;}}.$$

(6) Support system availability

The specific calculation sequence of support system availability is shown in Figure 10. First, according to the base LRU spare parts’ demand rate solved by the demand rate solving module and Formula (30), the single equipment demand rate is a function of the base LRU spare parts demand rate. So, the single equipment demand rate is obtained. Second, according to the base LRU spare parts support delay time solved by the spare parts demand–supply module and Formulas (31)–(33), the single equipment supply rate is a function of base LRU spare parts demand rate and support delay time. Therefore, the single equipment supply rate can be obtained according to the LRU spare parts demand rate and the support delay time. Then, according to Figure 15 and Formula (34), the base I system state steady-state probability is a function of single equipment demand rate and supply rate. It is the function of base LRU spare parts demand rate support delay time. Therefore, combining the above two, the base I system state steady-state probability can be obtained here. Next, according to Formula (36) and Figure 15, the base I equipment availability is a function of the base I steady-state probability in each system state. It is also a function of the base I LRU spare parts demand rate and support delay time. Therefore, the base I equipment availability can be obtained here. Finally, it can be seen from Formula (37) that the whole support system availability is a function of the base I equipment availability, which is also a function of the base LRU spare parts demand rate and support delay time. Therefore, the availability of the whole support system can be obtained from all bases’ equipment availability of the system.

### 3.7. Multi-Level Spare Parts Optimal Solution

The marginal analysis method [49,50,51,52] is used to solve the multi-level spare parts’ optimal mathematical model. Compared with other methods for solving mathematical models, such as the genetic algorithm [53,54] which is also widely used, the marginal analysis method has the advantages of a simpler calculation and solution process and stable solution results. Its optimal solution will not be lost. Every adjustment made by using the marginal analysis method to solve the spare parts inventory allocation strategy is the optimal solution under the current cost and availability. The optimal solution is also the initial spare parts inventory allocation state in the next iteration process.

#### 3.7.1. Optimization Mathematical Model

For equipment users, they will provide some support funds C_0_ of spare parts. They require the highest availability of no higher than spare parts funds C_0_, which is given by the optimal inventory allocation strategy. If there are spare parts inventory allocation strategies that meet the above constraints, this strategy is the optimal spare parts inventory allocation strategy. The strategy is provided to equipment users. According to the above description, the objective function is to maximize the support system availability. The constraint condition is that the cost of all spare parts is not greater than C_0_. Symbolizing the model is as in the following Equation (38):

(38) $$\left\{\begin{array}{c} max\;(S_{\mathrm{IJ}})A\\ s.t.C=\underset{I}{\sum }\underset{J}{\sum }S_{\mathrm{IJ}}\cdot C_{J} \end{array}\right.\leq C_{0}.$$

#### 3.7.2. Optimization Solution

First, a matrix s of m rows and n columns is constructed and initialized, that is, s = 0 (here, m represents the total quantity of all base and rear warehouse support points, n is the sum of all spare parts types).

Second, we traverse all positions of matrix s, calculate the marginal increment of each support point I and each type of spare part J. It is named as the following Equation (39):

(39) $$\Delta \mathrm{IJ}=\frac{A\left(S+\mathrm{ones}\left(I.J\right)\right)-A\left(S\right)}{C_{J}}.$$

Here, ones (I. J) is also m rows and n columns, which is the same type of matrix as matrix s. The value of row I and column J is 1, the rest are all 0. S + ones (I. J) means that a spare part inventory is added to the spare parts in the position of row I and column J. At this time, the spare parts of the selected (I. J) position are used as the ΔIJ spare parts inventory. The system availability increment of each RMB can be obtained when the inventory is added. When the spare parts inventory is s, the availability of the whole equipment support system A(S) is calculated according to the analytical model given in this paper.

Third, we select the maximum value in ΔIJ, add 1 to the value on position (I. J), to update the matrix S, S = S + ones (I. J) max.

**Figure 16:**
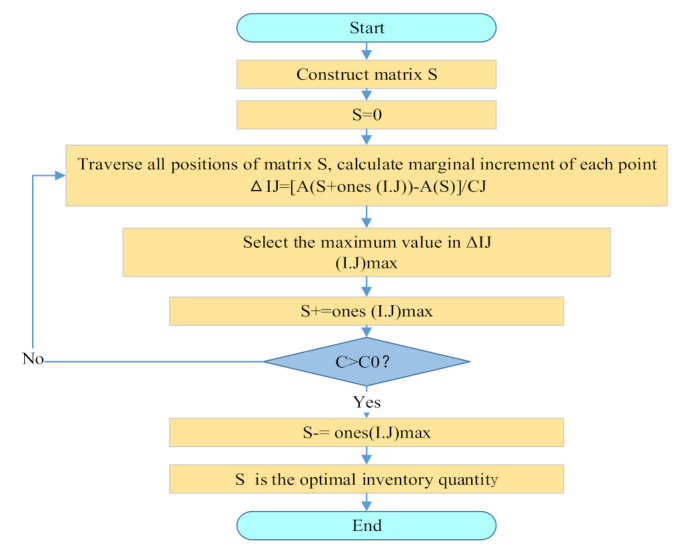
Flow chart for solving mathematical model of optimal inventory quantity.

Fourth, the total cost of the whole equipment support system spare parts with the updated matrix S is solved. The whole support system spare parts’ total cost is obtained as C. The system spare parts’ total cost corresponding to the updated matrix C is compared with the funds C_0_ given by the equipment users. If C > C_0_ is true, the new matrix s updated at this time is regressed. That is, S = S- max (ones (I.J)). At this time, when the updated matrix s goes back one step, the spare parts’ various types of configuration quantity corresponding to the generated matrix s is the optimal inventory quantity we want. The model solution ends. If C > C_0_ is false, we go to step (2) and continue the iterative solution. The specific solution process is shown in Figure 16.

## 4. Model Simulation and Verification

### 4.1. Simulation Model

**Figure 17:**
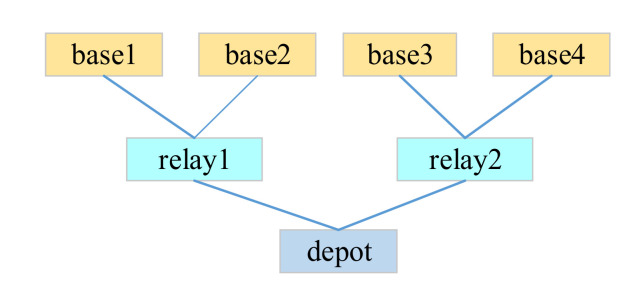
Three-level equipment support system.

A simulation model, which is the reverse process of the demand–supply steady-state process-based multi-level spare parts support process is designed in the paper. The availability solution method in the simulation model is derived from the spare parts support optimal model. Secondly, the assumed data are substituted into the analytical model. The spare parts inventory and the availability optimal solution under the current situation are obtained after the corresponding iteration multiplies the lower availability bound. Then, the optimal solution is substituted into the simulation model to obtain the simulation model availability. Then, the two kinds of availability are compared to demonstrate the analytical model’s feasibility. Finally, the analytical model is compared with VMETRIC software to validate model accuracy in a low system availability bound. The analytical model applicability is obtained. The simulation model is implemented on MATLAB. For the convenience of the description, the simulation model assumes that the equipment is composed of LRU and SRU and the spare parts support system is a three-level support system, which is the base level, intermediate station level and the rear warehouse level, as in Figure 17.

**Figure 18:**
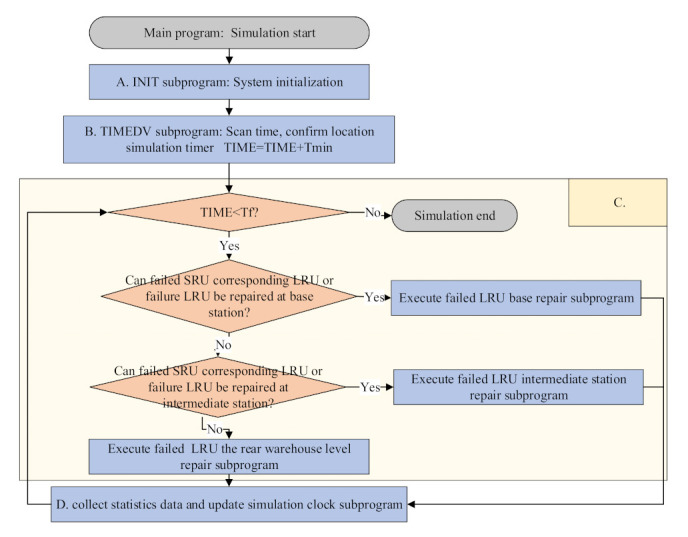
Simulation program’s structure flow chart.

According to the SRU or LRU failure, the simulation process can work. The simulation model driving events can be specifically described as all LRU and SRU failure events. According to the sequence of occurrences, the system can carry out level-by-level maintenance activities for the corresponding failure of spare parts in the equipment. The specific overall simulation structure is shown in Figure 18.

The simulation program’s overall structure is mainly composed of ABCD four parts. The main program is composed of six subroutines, which are INIT subroutine, TIMEDV subroutine, failure LRU base maintenance subroutine, failure LRU intermediate level maintenance subroutine, failure LRU rear warehouse maintenance subroutine, statistical data collection and simulation clock updating subroutine. The four parts are described as follows.

A is the init program, which is the system initialization. First, the program gives the current simulation initial clock time = 0, and simulation end clock T_f_. T_f_ is a simulation support cycle. Then, the program makes the downtime of all equipment deployed in the base Td_IN_ = 0 (n = 1, 2, …, N_I_). The subscript IN denotes the equipment marked n in base I.

B is the TIMEDV program. The TIMEDV program scans the event table to determine the current simulation clock time. It scans all the events that do not occur and finds the time T^min^ of the earliest event, so that time = time + T_min_. The events not occurring here refer to the MTBF all SRUs or LRUs that have not failed.

C is the main program process. First, the program judges whether the current simulation time is less than the simulation end time. That is whether TIME < T_f_ is true. If it is true, it means that the current simulation program can still be carried out. Then it judges whether the current event can be repaired at the base level. That is whether the corresponding LRU failure is caused by the failed SRU or whether the direct LRU failure can be repaired at the base level. The corresponding base level failure LRU maintenance program is executed. The time caused by maintenance and transportation is recorded. D program continues to execute after executing the base-level maintenance program. If the LRU failure or direct failure LRU caused by the failed SRU cannot be repaired at the base level, it will be transported to the intermediate station level for repair. If the intermediate station can repair, the corresponding intermediate station level failed LRU repair program will be executed. The time caused by the repair and transportation will be recorded too. After the intermediate station level repair program is executed, the D program will continue to be executed. If the failed LRU cannot be repaired at the base level or the intermediate station level, it is repaired at the rear warehouse level. The rear warehouse level repair program is executed. The maintenance and transportation time is recorded. Because we have assumed that our rear warehouse has unlimited maintenance capacity, all failed spare parts can be successfully repaired at the rear warehouse level. After executing the rear warehouse level maintenance program, continue to execute the D program.

D collects statistics data and updates the simulation clock program. First, Statistics data are collected in the program. The downtime of all equipment due to maintenance failure LRU is td_in_ = td_in_ + T_repair_ + T_transportation_. The T_repair_ is the corresponding time of handling the event in C, including the maintenance time and the transportation time of spare parts from the base to the corresponding maintenance point. T_transportation_ is the transportation time of the repaired parts from the maintenance point to the equipment base. Second, the simulation time is updated. TIME = TIME + T_repair_ + T_transportation_. It finds the next fault event, which is to ensure that the equipment can work normally again.

The A, B, C and D parts’ complete execution is the end of simulation execution. To ensure the stability of the simulation results, we should ensure that the simulation end clock T_f_ and the total simulation times are as large as possible. After the whole simulation, the corresponding downtime due to maintenance failure spare parts and transportation spare parts is stored in the Td_IN_ of each piece of equipment. Therefore, the unavailability of the equipment is Td_IN_/T_f_. The corresponding availability is 1-Td_IN_/T_f_. All equipment availability A_Isim_ obtained by this simulation model in base I can be shown in Equation (40).

(40) $$A_{\mathrm{Isim}}=1-\sum_{N=1}^{N_{I}}\left(1-\frac{{\mathrm{Td}}_{\mathrm{IN}}}{T_{f}}\right).$$

The total availability $A_{\mathrm{sim}}$ of the whole support system b bases is shown in Equation (41).

(41) $$A_{\mathrm{sim}}=\frac{\sum_{I=1}^{b}\left(A_{\mathrm{Isim}}\cdot N_{I}^{\;}\right)}{\sum_{I=1}^{b}N_{I}^{\;}}.$$

### 4.2. Experiment Verification

We suppose that the spare parts’ support system consists of three levels of support system. It includes base level, intermediate site level and rear warehouse level. There are four base levels, two relay levels and one depot level, and their specific relationship is shown in Figure 17. The equipment multilayer spare parts are composed of LRU and SRU. The specific composition structure is shown in Figure 3. For the four bases, the numbers of equipment configurations are shown in Table 3. Table 4 shows the transportation time between stations of each grade.

For spare parts, LRU and SRU, the failure maintenance time, unit price, the numbers of single machine assembling, repair probability and repair time at each maintenance level are shown in Table 5 and Table 6. In Table 5, r_1j_ represents the probability that the spare parts can be repaired in base 1. The r_2j_ represents the probability that the spare parts can be repaired in base 2. The r_3j_ is the probability that the spare parts can be repaired in base 3. The r_4j_ is the probability that the spare parts can be repaired in base 4. The r_R1j_ is the probability that the spare parts can be repaired in intermediate site 1. The r_R2j_ is the probability that the spare parts can be repaired in intermediate site 2. Because we assume that the system maintenance capability is unlimited and the rear warehouse default repair probability is 1. In Table 6, The R1j is the maintenance time of corresponding spare parts in base 1. The R_2j_ is the maintenance time of corresponding spare parts in base2. The R_3j_ is the maintenance time of corresponding spare parts in base 3. The R_4j_ is the maintenance time of corresponding spare parts in base4. The R_R1j_ is the maintenance time of corresponding spare parts in intermediate station 1. The R_R2j_ is the maintenance time of corresponding spare parts in intermediate station 2. R_0j_ is the maintenance time of corresponding spare parts in the rear warehouse. To simplify the calculation, we assume that the same spare part’s maintenance probability and maintenance time at different maintenance sites at the same level are the same.

We set the equipment system’s minimum availability as 90%, 95% and 98%. Firstly, these parameters are calculated in the multi-echelon and multi-indenture spare parts optimization model. The analytical solution is obtained. Then, we substitute these analytical solutions into our simulation model to get the corresponding availability and then evaluate our analytical model.

By substituting the input parameters into the spare parts’ optimization analytical model, we can get the result that when the marginal analysis method iterates 57 times, the first availability is greater than 90%. So, the spare parts’ quantity is the optimal spare parts’ inventory under this condition. At this time, the availability is 90.36% and the spare parts’ total cost of the whole support system is 6.1 million RMB. The specific spare parts’ configuration scheme is shown in Table 7.

Then, the lower availability bound is set to 95% and 98% for analysis. In the analytical model, the 95% lower availability bound is 15 iterations based on 90% lower availability and 57 iterations. It is 72 iterations. The lower 98% availability bound is nine iterations based on 95%. It is 81 iterations. The optimal inventory schemes of 95% lower availability bound and 98% lower availability bound are shown in Table 8 and Table 9.

**Figure 19:**
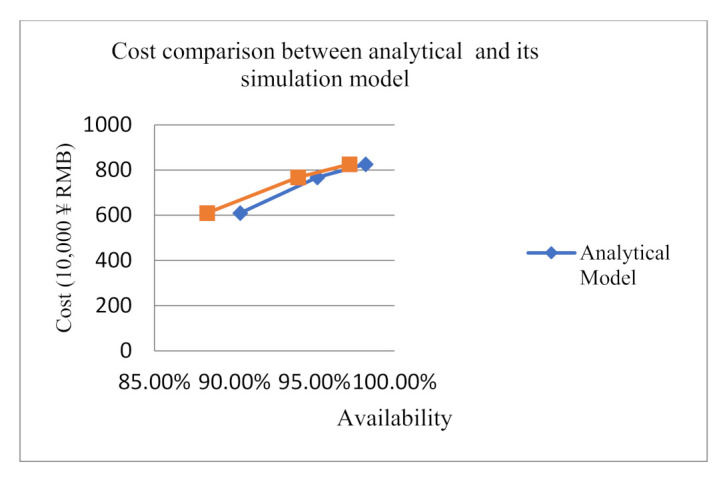
Comparison between analytical model and simulation model.

By substituting the three optimal inventory quantities of spare parts of the analytical model into the simulation model designed in the paper, the simulation availability is 88.29%, 93.97% and 97.19%. The specific data are shown in Table 10 and Figure 19. It can be seen from the comparison results that the availability of the analytical model is slightly higher than that of the simulation model. However, the relative error is within the acceptable range.

**Figure 20:**
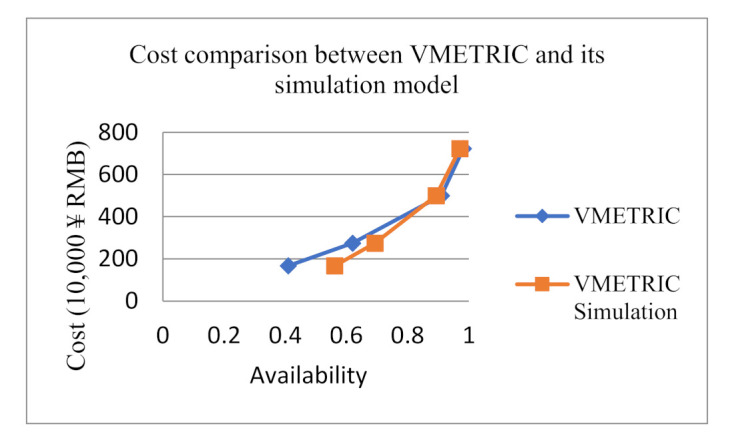
Comparison VMETRIC model and its simulation model.

**Figure 21:**
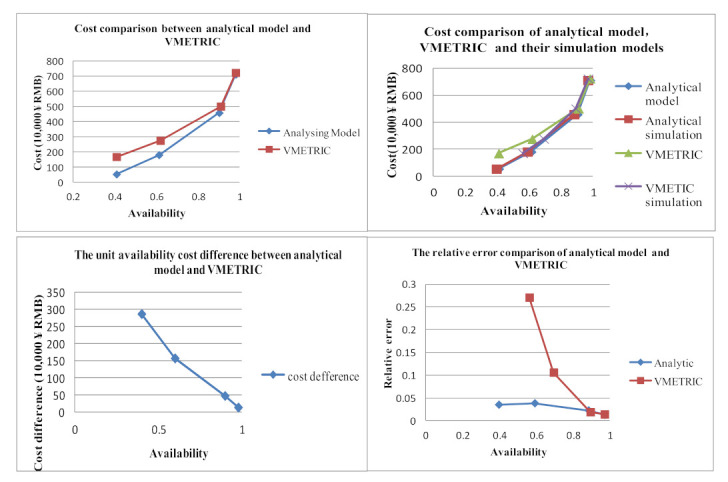
Comparison between analytical model and VMETRIC.

To further verify the rationality of the inventory plan obtained by our analytical model, this paper compares the results of the VMETRIC software in Reference [13]. The assumptions and input parameters refer to the literature [13]. Here, we also input the data of the literature, and the comparison results are shown in Table 11. The unit availability cost difference = (VMETRIC software cost—analytic model cost)/current availability lower bound. This variable is defined here to represent the advantage of the analytical model over VMETRIC software from the cost perspective. The cost comparison between the VMETRIC model and its simulation model is shown in Figure 20. The cost comparison, unit availability cost difference and relative error between the analytical model and the VARI-METRIC model are shown in Figure 21.

### 4.3. Result Analysis

It can be seen from the comparison results that the availability of the analytical model is slightly higher than that of the simulation model, which is shown in Table 5, Table 6, Table 7 and Table 8 and Figure 19. However, the relative error is within the acceptable range. When the availability bound is larger, the relative error is smaller. So as long as we have enough funds, our model will be more accurate. After many other input parameters and other lower availability bounds calculations, it is found that the results are approximately consistent with ours. Our analytical model has errors compared with the simulation model. By analyzing the reasons, we can find that we have made a lot of assumptions in the model analysis process. These assumptions will lead to errors in the solution of the analytical model and the simulation model. However, from the results analysis, we find that the availability obtained by our analytical model can generally meet the requirements of equipment system support.

From Table 9 and Figure 21, we can conclude that the accuracy of the spare parts inventory prediction of the analytical model is higher than that of the VARI-METRIC model under the condition of low system availability.

First, when the low availability abound of equipment system is 0.4, 0.6, 0.9, 0.98, the relative error between analytical model and simulation model is 3.54%, 3.86%, 2.29%, 1.29%, the relative error between VARI-METRIC model and its simulation model is 27.06%, 10.60%, 1.88%, 1.14%. Comparing the relative error of availability between the analytical model and VMETRIC software, it is found that the accuracy of the analytical model is higher than that of VMETRIC software when the lower availability bound is small. Its relative error is less than 4% when the availability lowers to 60%. However, VARI-METRIC’s relative error is greater than 10%. However, when the lower availability bound is large enough, the VARI-METRIC accuracy is approximately consistent with that of the analytical model. Even the VARI-METRIC accuracy is a little higher than that of the analytical model.

Second, when the low abound availability of equipment system is 0.4, 0.6, 0.9, 0.98, the cost of the analytical model is 0.52, 1.795, 4.575, 7.11 million ¥ RMB, and the cost of the VMETRIC model is 1.67, 2.74, 5.00, 7.23 million ¥ RMB. The price difference between the analytical model and VARI-METRIC model is 2.875, 1.575, 0.4722, 0.1333 million ¥ RMB. Comparing the cost of the analytical model with VMETRIC software and the cost difference of comprehensive unit cost availability, it can be found that the cost of the analytical model is lower than that of VMETRIC software in the case of any lower availability bound. However, with the increase of the lower availability bound, the cost advantage of the analytical model gradually weakens until it can be ignored. The reason is that there are two key approximations in the algorithm of the VARI-METRIC model. First, using the product of LRU availability to calculate equipment availability will lead to low results. Second, the impact of equipment downtime is not considered in the calculation of demand rate, which estimates a demand rate higher than the actual one, and will also lead to lower availability results.

For the VARI-METRIC model being realized by VMETRIC software, our comparison between the analytical model and VMETRIC software is suitable for the VARI-METRIC model and analytical model. So, the analytical model solves the accuracy problems of the VARI-METRIC model with a low equipment system availability bound. With high availability bound, the analytical model cost advantage is not obvious. When we care about support cost and a small quantity of availability error, the analytical model has some superiority. Therefore, the analytical model proposed in this paper can be applied to a wider range.

To ensure the reliability of the equipment system and reduce the cost of spare parts inventory, some management suggestions are as follows. First, it is necessary to establish a reasonable maintenance supply support system and make hierarchical support equipment available. Secondly, adhere to the standardization of spare parts’ use, realize the sharing of spare parts, and reduce the maintenance queue and inventory cost of spare parts. Third, according to the spare parts inventory allocation strategy and inventory situation, specify a scientific spare parts procurement plan and supply reasonably and orderly. Fourth, strengthen the repair and reuse of spare parts. Finally, the maintenance capacity of maintenance support stations at all levels shall be provided as much as possible.

## 5. Conclusions

This paper proposes the demand–supply steady-state process-based multi-level spare parts optimization model. It is based on a multi-echelon maintenance supply and multi-indenture equipment support process. The marginal analysis method [42,43] is used to solve the multi-level spare parts’ optimal mathematical model. This paper designs the three-level equipment (equipment system, LRU, SRU) and the two-level maintenance supply model (base and rear warehouse) to verify the consistency between the analytical model and the simulation model designed according to the analytical model. Then, the paper makes a comparative experiment between the analytical model and the VARI-METRIC model with the data from Reference [13]. The simulation results show that when the availability of equipment system is 0.4, 0.6, the relative error of the analytical model is 3.54%, 3.86%, and the relative error of the VARI-METRIC model is 27.06%, 10.60%, the cost of the analytical model is 0.52, 1.795million ¥ RMB, and the cost of the VARI-METRIC model is 1.67, 2.74, million ¥ RMB. When the availability of the equipment system is 0.9, 0.98, the relative error of the analytical model is 2.29%, 1.29%, and the relative error of the VARI-METRIC model is 1.88%, 1.14%, the cost of the analytical model is 4.575, 7.11 million ¥ RMB, and the cost of the VARI-METRIC model is 5.00, 7.23 million ¥ RMB. When the system availability is low, the inventory prediction accuracy of the analytical model is significantly higher than that of the VARI-METRIC model. When the system availability is high, the inventory prediction accuracy of the analytical model is basically equivalent to that of the VARI-METRIC model. Therefore, the analytical model proposed in this paper improves the prediction accuracy of the spare parts inventory strategy of the VARI-METRIC model under the condition of low system reliability. The model accuracy is verified to meet the requirements of equipment support. It can be applied to a wider range.

Whether the analytic model proposed in this paper or the VARI-METRIC model, some approximate processing methods are adopted in the modeling process, which cannot guarantee accurate or even correct results in any case. To simplify the model calculation, some restrictions proposed in this paper lead to some limitations of this study. Limitations of this study: First, this model assumes that the maintenance probability of spare parts in the rear warehouse is 1 and there are no scrap parts, but there is a lack of maintenance capacity in the rear warehouse and scrap spare parts. Second, this model assumes that only one SRU can cause one LRU failure. However, in reality, multiple SRUS may cause LRU failure. Third, this model assumes that all spare parts at the same level are in a series relationship. The spare parts of complex equipment are in a series-parallel relationship. Therefore, our further research work will consider the LRU failure caused by waste parts in the rear warehouse and multiple SRU parts. At the same time, the relationship between spare parts will be extended from a series relationship to a series-parallel hybrid relationship. The maintenance channel of the support level station is changed from an unlimited maintenance channel to a limited maintenance channel. In the future, some new intelligent technologies, such as machine learning and RFID [9], will be used to build the equipment support system, improve the calculation method of the model, improve the accuracy of the inventory allocation strategy and improve the equipment support capability.

## Figures and Tables

**Table 1:** The advantages and disadvantages of optimization models.

Optimization Models	Description	Advantage	Disadvantage
METRIC [14,26,27]	The basic model of multi-level spare parts optimization solves the problem of optimizing the performance of a Multi-level Inventory System under the specified investment level of the inventory system.	Solve the problem of optimizing the performance of the inventory system under the specified investment level of the inventory system.	The ordering cost of final products, maintenance channels, assembly repair of series parts, horizontal supply, etc. is not considered. The system availability is not considered enough.
Mod-METRIC [25,27,28]	A cost-minimization model for expected delayed ordering of final products with multiple spare parts and orders.	Solve the problem of minimizing the expected delay order cost of the final product.	Maintenance channels, serial-parallel repair and horizontal supply were not considered.
Dyna-METRIC [25,29,30,49]	The instantaneous activity dynamic inventory model of spare parts delivery and inventory system.	A dynamic inventory model considering wartime unsteady operational requirements and spare parts support decision-making.	It ignores the maintenance support level, resulting in the maintenance of components that may not be those that can increase the available quantity most Priority scheduling inducts the component causing the most unavailable aircraft.
VARI-METRIC [25,31,33,34,35,36,37,38,39,40,41,42,43,44,45,46,47,48]	At present, the final form used is a multi-level support structure and multi-level spare parts optimization model.	It solves the optimization problem of multi-level spare parts under the conditions of limited maintenance channel, series repair, horizontal supply etc. It is suitable for high system availability. It is widely used in complex equipment, such as aircraft, missiles, submarines, etc.	Many assumptions are made in the process of establishing the model, which leads to the low accuracy of the equipment system in low availability. The relative error of equipment system is large under low availability of the equipment system.

**Table 2:** Definitions of spare parts optimization parameters.

Parameters	Definition	Units	Parameters	Definition	Units
MTBF_J_	SRU_J_ average fault interval	hour	r_IJ_	a probability that SRU_J_ failure can be repaired at base I	
q_IJ_	LRU repaired at base I is the probability caused by failure, where ∑(J = 1,n = 1) q_IJ_ = 1		T_J_	the time from SRU_J_ application to delivery for ordering application from the back warehouse	hour
S_IJ_	SRU_J_ inventory of base I	piece	Z_J_	the SRU_J_ number of single machine installations	piece
dT_IJ_	SRU_J_ average shortage time of base I	hour	C_J_	spare parts SRU_J_ unit price	¥ RMB
EBO_IJ_	SRU_J_ expected shortage of base I	hour	N_I_	the number of equipment deployed in base I	piece
λ_IJ_	SRU_J_ demand rate of base I		WT_I_	average daily working time of equipment in base I	hour
*V*_IJ_	SRU_J_ supply rate of base I under single maintenance channel		A_I_	availability of equipment at base I	
PS_IJ_	SRU_J_ the steady probability of base I when the inventory is S		R_IJ_	SRU_J_ average maintenance time for the base I	hour

**Table 3:** Spare parts allocation quantity.

Spare Parts	Base1	Base2	Base3	Base4
number	5	5	2	2

**Table 4:** Schedule of application for supply transportation.

Start to End	Time (Day)	Start to End	Time (Day)
depot-relay1	10	depot-relay2	9
relay1-base1	8	relay2-base1	7
relay1-base2	6	relay1-base2	5

**Table 5:** Input parameters of each spare part.

Spare Parts	MTBF (h)	C (¥ RMB)	Z	r_1j_	r_2j_	r_4j_	r_3j_	r_R1j_	r_R2j_
LRU1	1000	100,000	3	0.5	0.5	0.5	0.5	0.7	0.7
LRU2	2000	200,000	2	0.55	0.55	0.55	0.55	0.75	0.75
LRU3	3000	300,000	1	0.6	0.6	0.6	0.6	0.8	0.8
SRU11	1000	10,000	3	0.4	0.4	0.4	0.4	0.6	0.6
SRU12	1500	20,000	3	0.3	0.3	0.3	0.3	0.5	0.5
SRU21	2000	30,000	2	0.45	0.45	0.45	0.45	0.65	0.65
SRU22	2500	40,000	2	0.35	0.35	0.35	0.35	0.55	0.55
SRU31	3000	50,000	1	0.5	0.5	0.5	0.5	0.7	0.7
SRU32	3500	60,000	1	0.4	0.4	0.4	0.4	0.6	0.6

**Table 6:** Input parameters of each spare part.

Spare Parts	R_1J_	R_2J_	R_3J_	R_4J_	R_R1J_	R_R2J_	R_0J_
LRU1	5	5	5	5	7	7	9
LRU2	6	6	6	6	8	8	10
LRU3	7	7	7	7	9	9	11
SRU11	3	3	3	3	5	5	7
SRU12	4	4	4	4	6	6	8
SRU21	4	4	4	4	6	6	8
SRU22	5	5	5	5	7	7	9
SRU31	5	5	5	5	7	7	9
SRU32	6	6	6	6	8	8	10

**Table 7:** Optimal spare parts inventory and general evaluation with 90% availability lower bound.

Spare Parts	Base1	Base2	Base3	Base4	Relay1	Relay2	Depot
LRU1	3	4	1	2	0	1	0
LRU2	2	3	1	1	1	2	1
LRU3	1	2	0	1	1	1	0
SRU11	0	1	0	1	1	1	1
SRU12	1	0	1	1	0	1	1
SRU21	0	1	1	0	1	0	1
SRU22	1	1	0	1	1	1	2
SRU31	0	0	1	0	1	0	1
SRU32	1	1	0	0	1	1	1
Iteration number	57						
availability	90.36%						
Total cost (¥ RMB)	6,100,000						

**Table 8:** Optimal spare parts inventory and general evaluation with 95% availability lower bound.

Spare Parts	Base1	Base2	Base3	Base4	Relay1	Relay2	Depot
LRU1	4	5	2	2	1	1	0
LRU2	3	4	1	1	1	2	1
LRU3	2	2	1	1	1	1	0
SRU11	0	1	1	1	1	2	1
SRU12	1	1	1	1	1	1	2
SRU21	1	1	1	0	1	1	1
SRU22	1	1	0	1	1	1	2
SRU31	0	0	1	0	1	0	1
SRU32	1	1	0	0	1	1	1
Iteration number	72						
availability	95.17%						
Total cost (¥ RMB)	7,680,000						

**Table 9:** Optimal spare parts inventory and general evaluation with 98% availability lower bound.

Spare Parts	Base1	Base2	Base3	Base4	Relay1	Relay2	Depot
LRU1	5	5	2	3	1	1	0
LRU2	3	4	1	2	1	2	1
LRU3	2	2	1	1	1	1	0
SRU11	1	1	1	1	1	2	2
SRU12	1	1	1	1	1	1	2
SRU21	2	1	1	0	1	1	1
SRU22	1	1	0	1	1	1	1
SRU31	0	1	1	0	1	1	1
SRU32	1	1	0	1	1	1	1
Iteration number	81						
availability	98.20%						
Total cost (¥ RMB)	8,260,000						

**Table 10:** Comparison between analytical model and simulation model.

Availability Lower Bound	Iteration Number	Cost (¥ RMB)	Availability	Availability Simulation	Relative Error
90%	57	6,100,000	90.36%	88.29%	2.34%
95%	72	7,680,000	95.17%	93.97%	1.28%
98%	81	8,260,000	98.20%	97.19%	1.04%

**Table 11:** Comparison between the analytical model and VMETRIC software.

Abound	Analytical Model				VMETRIC [13]				Cost Difference (Million ¥ RMB)
	A	Asim	C (Million ¥ RMB)	Relative Error	A	Asim	C (Million ¥ RMB)	Relative Error	
40%	40.93%	39.53%	0.52	3.54%	40.94%	56.13%	1.67	27.06%	2.875
60%	61.32%	59.04%	1.795	3.86%	61.98%	69.33%	2.74	10.60%	1.575
90%	90.27%	88.25%	4.575	2.29%	91.01%	89.33%	5.00	1.88%	0.4722
98%	98.22%	96.97%	7.11	1.29%	98.17%	97.06%	7.23	1.14%	0.1333

## Data Availability

The data can be accessed from this manuscript. The VMETRIC model input data used for comparison in paper is from reference [10].
